# Identification and Characterization of microRNAs during Retinoic Acid-Induced Regeneration of a Molluscan Central Nervous System

**DOI:** 10.3390/ijms19092741

**Published:** 2018-09-13

**Authors:** Sarah E. Walker, Gaynor E. Spencer, Aleksandar Necakov, Robert L. Carlone

**Affiliations:** Department of Biological Sciences, Brock University, St. Catharines, ON L2S 3A1, Canada; sw10tn@brocku.ca (S.E.W.); gspencer@brocku.ca (G.E.S.); anecakov@brocku.ca (A.N.)

**Keywords:** retinoic acid, microRNA, RNA sequencing, neuronal regeneration, growth cone, *Lymnaea*

## Abstract

Retinoic acid (RA) is the biologically active metabolite of vitamin A and has become a well-established factor that induces neurite outgrowth and regeneration in both vertebrates and invertebrates. However, the underlying regulatory mechanisms that may mediate RA-induced neurite sprouting remain unclear. In the past decade, microRNAs have emerged as important regulators of nervous system development and regeneration, and have been shown to contribute to processes such as neurite sprouting. However, few studies have demonstrated the role of miRNAs in RA-induced neurite sprouting. By miRNA sequencing analysis, we identify 482 miRNAs in the regenerating central nervous system (CNS) of the mollusc *Lymnaea* *stagnalis*, 219 of which represent potentially novel miRNAs. Of the remaining conserved miRNAs, 38 show a statistically significant up- or downregulation in regenerating CNS as a result of RA treatment. We further characterized the expression of one neuronally-enriched miRNA upregulated by RA, *miR-124*. We demonstrate, for the first time, that *miR-124* is expressed within the cell bodies and neurites of regenerating motorneurons. Moreover, we identify *miR-124* expression within the growth cones of cultured ciliary motorneurons (pedal A), whereas expression in the growth cones of another class of respiratory motorneurons (right parietal A) was absent in vitro. These findings support our hypothesis that miRNAs are important regulators of retinoic acid-induced neuronal outgrowth and regeneration in regeneration-competent species.

## 1. Introduction

Following injury to the central nervous system (CNS), the ability of damaged neurons to repair and regenerate functional connections is limited in most species. Only a few vertebrates are able to regenerate lost or damaged CNS tissues and cell types, though numerous invertebrates possess this intrinsic ability of self-repair [1,2]. Interestingly, many trophic and chemotropic factors that mediate neuronal outgrowth and connectivity in regeneration-competent species are highly conserved and functional in many vertebrates and invertebrates [3,4,5]. However, a thorough understanding of the molecular and biochemical mechanisms underlying the production and utilization of these factors that results in functional CNS regeneration is lacking.

One critical factor that has been suggested to play a role in CNS regeneration in many species is the vitamin A metabolite, all-*trans* retinoic acid (RA). RA signaling mediates neuronal outgrowth [6] and differentiation [7] during both CNS development and regeneration [8,9,10]. To exert such effects, RA binds to two classes of nuclear receptors, the retinoic acid receptors (RAR) and the retinoid X receptors (RXR). Following ligand binding, specific subtypes of these receptors typically heterodimerize to regulate the expression of genes that contribute to numerous processes associated with both neuronal development and regeneration [7]. Our understanding of the role of RA signaling in CNS regeneration has come largely from studies on urodele amphibians, such as the newt and axolotl [9,11,12,13]. More recently, the role of specific microRNAs as post-transcriptional regulators, both upstream and downstream of RA signaling in the regenerating CNS, has become an important area of investigation.

MicroRNAs (miRNAs) are conserved, non-coding RNAs that post-transcriptionally regulate gene expression by directly binding to mRNAs to suppress their translation into functional proteins. In the adult newt, *miR-133a* was identified by microarray analysis as being a critical regulator of *RARβ* expression. [14,15]. When the expression of this miRNA was experimentally increased by electroporation of a mimic, RARβ expression was downregulated and posterior ependymal tube outgrowth from the cut spinal cord was inhibited [14]. Moreover, *miR-133* has also been implicated in the regulation of spinal cord regeneration in zebrafish [16]. However, no studies to date have examined the role of miRNAs in RA-mediated CNS regeneration in any invertebrate species.

RA signaling was originally thought to be a vertebrate innovation, but has now been demonstrated in some CNS regeneration-competent invertebrate species [17], such as the pond snail, *Lymnaea stagnalis*. RA is found in the CNS of *Lymnaea stagnalis* [17], and induces neurite sprouting and attractive growth cone turning in various neuronal cell types [10,18]. Moreover, components of the retinoid signaling pathway, including RAR and RXR, are expressed in the growth cones and mediate RA-induced chemotropic responses [19,20]. As *Lymnaea* CNS neurons can regenerate and this can be enhanced by RA signaling, our aim here was to determine whether miRNAs play a role in the regenerative response of this invertebrate CNS.

In this study, we identified miRNAs in the regenerating CNS of adult *Lymnaea* and discovered a specific subset that were either upregulated or downregulated during RA-induced neurite sprouting. We then examined the spatial and temporal patterns of expression of one of these dysregulated miRNAs, *miR-124*. Interestingly, we found that *miR-124* displayed similar expression patterns in *Lymnaea* as it does in many vertebrates. Specifically, we found *miR-124* was enriched in the adult CNS, and was upregulated during both development and CNS regeneration. Using in situ hybridization, we also examined the spatial distribution of *miR-124* in regenerating motorneurons, and demonstrated a cell-specific expression in regenerating growth cones.

## 2. Results

### 2.1. miRNA Sequencing Analysis of miRNAs in the Lymnaea Central Nervous System (CNS)

As RA is capable of inducing neurite outgrowth from both vertebrate and invertebrate nerves, the identification and functional characterization of miRNAs that might be involved in mediating such retinoid-induced outgrowth was our primary focus. As a model system, we utilized adult CNS from the regeneration-competent mollusc, *Lymnaea stagnalis*, whereby neurite sprouting from cut nerves, as well as from individually cultured neurons, were examined. 

**Figure 1:**
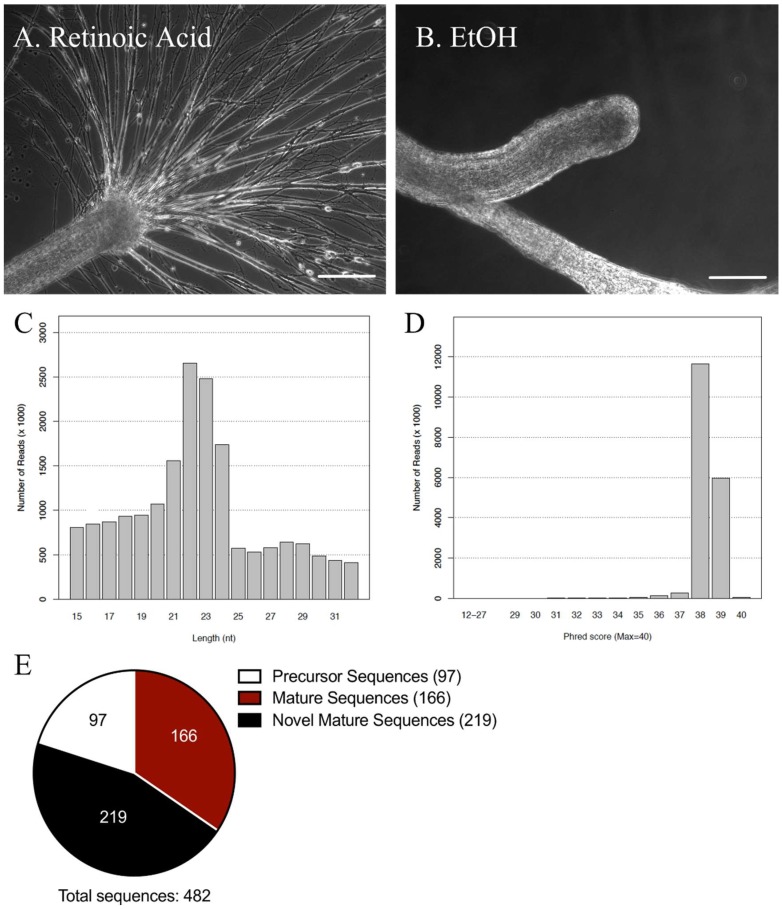
miRNA sequencing data of regenerating *Lymnaea* central nervous system (CNS). (**A**,**B**) Representative images of regenerative outgrowth from cut nerves emerging from the *Lymnaea* CNS. Robust neurite outgrowth is seen in CNS incubated in retinoic acid (RA) (**A**), though no outgrowth is seen from CNS incubated in EtOH (vehicle control) (**B**); Scale bars = 250 μm; (**C**) Quality assessment of miRNA sequencing analysis using the length and count distribution of sequences. Graph depicts the length distribution of reads following the 3′adapter cut. Counts display a majority of reads in the range of 21–24 nucleotides, corresponding to the length of mature miRNAs; (**D**) Histogram of the average Phred score per base in a read after the 3′adapter cut. A Phred score greater than 30 corresponds to an accuracy of 99.9%, while a score of 40 represents an accuracy of 99.99%; (**E**) Number of novel, mature, and precursor miRNAs identified in the *Lymnaea* CNS.

The CNS of *Lymnaea* was removed from the animal by transecting all nerves emanating from the CNS to the periphery. The CNS was incubated in either RA (10^−5^ M) or EtOH (0.1%; vehicle control) for 72 h, which was sufficient time for a regenerative response to occur. We found that when dissected CNS were incubated in RA, the nerves exhibited robust neurite sprouting (Figure 1A), which did not occur in CNS incubated in EtOH alone (Figure 1B). Total RNA was isolated from pooled samples of both RA-treated (regenerating/sprouting) and EtOH-treated (non-regenerating) tissues 72 h following treatment, and submitted for miRNA sequencing analysis.

The quality of our miRNA sequencing analysis was validated with appropriate length and count distributions of the sequences and optimal Phred scores, to ensure accuracy of the reads (Figure 1C,D). The length distribution (Figure 1C) determined that the average length of our mappable reads was ~22–23 base pairs, which is representative of the typical length of mature miRNAs [21]. The Phred scores identified the base call accuracy, and determined the probability of an incorrect base reading. A perfect score of 40 corresponds to 99.99% accuracy of the reads, while a score greater than 30 corresponds to 99.9% accuracy. Our data displayed typical Phred scores of 38 and 39 (Figure 1D), suggesting a high accuracy (>99.9%) and providing confidence in our miRNA sequencing data analysis.

Mature miRNA sequence information in molluscs is currently very limited. At present, miRBase release 22 (March 2018; available online: http://www.mirbase.org/) reports the sequences of only three molluscan species, *Lottia gigantea*, *Melibe leonina*, and *Haliotis rufescens*. To identify miRNAs in *Lymnaea*, the sequences generated from our miRNA sequencing analysis were matched to known miRNAs in the mollusc, *Lottia gigantea*. The miRNA sequencing analysis identified 482 miRNA sequences in the *Lymnaea* CNS, including 97 precursor miRNAs, 166 mature miRNAs and 219 novel miRNAs (Figure 1E; See Supplementary Material for list of all identified miRNA sequences). The novel miRNAs represent a group of mature miRNAs that were not matched to any other known species in miRBase. As most miRNAs are highly conserved, even between distantly related species such as invertebrates and vertebrates [22], these novel miRNA sequences may be *Lymnaea*-specific miRNAs, or possibly, miRNAs that have not yet been identified or sequenced in other species. It is also possible that some of these novel miRNAs represent RNA fragments.

### 2.2. Identification of miRNAs That Were Differentially Regulated during Retinoic Acid (RA)-Induced Regeneration

**Figure 2:**
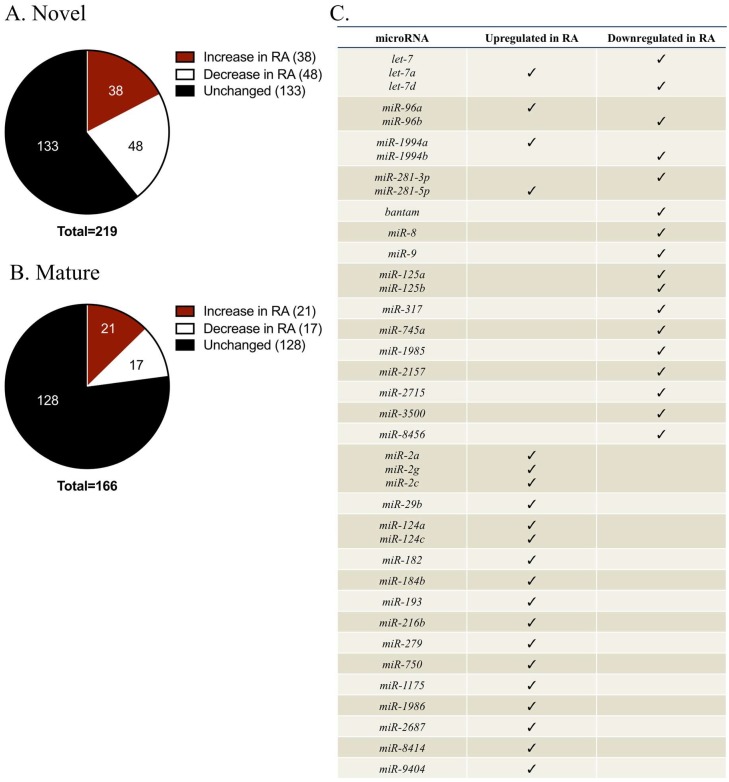
Mature miRNAs that were differentially expressed between regenerating CNS incubated in RA, or non-regenerating CNS incubated in EtOH; (**A**) Pie chart depicts the total number of novel sequences identified by miRNA sequencing. Of these sequences, 38 exhibited at least a 2-fold increase in CNS incubated in RA (red), while 48 displayed at least a 2-fold reduction in RA-treated samples (white); (**B**) Pie chart depicts the total number of mature miRNA sequences identified by miRNA sequencing analysis. Of the identified sequences, a large proportion did not exhibit differential expression between CNS incubated in RA and EtOH (black). However, a small subset exhibited at least a 2-fold change between treatment groups, and were either upregulated (red) or downregulated (white) in RA-treated CNS; (**C**) A complete list of differentially expressed mature miRNAs following RA treatment. Table indicates the mature miRNA name and its corresponding expression pattern in regenerating RA-treated CNS.

From our miRNA sequencing data, 219 putative novel sequences represented a large proportion of total sequenced reads (~45%). Of these novel sequences, 38 exhibited at least a 2-fold increase in RA-treated samples, while 48 displayed at least a 2-fold reduction (Figure 2A). However, all of the differentially regulated novel sequences were of extremely low abundance, typically with less than 10 sequence reads in the entire CNS. Due to their extremely low level of expression, we did not investigate any novel sequences further. 

We next chose to focus on mature miRNAs that had previously been identified in other species. Of the 166 mature miRNAs, 21 sequences exhibited at least a 2-fold increase in RA-treated CNS, while 17 miRNAs displayed at least a 2-fold reduction (Figure 2B,C). A literature examination of many of these differentially regulated miRNAs shed some light on their known roles, and many were found to contribute to the formation and/or function of the nervous system (Table 1). Many of these miRNAs also exhibited overlapping cellular functions, contributing to processes such as tumorigenesis [23,24,25,26,27,28,29,30], differentiation [31,32,33], proliferation [34,35,36,37,38], apoptosis [39,40,41,42], and cell cycle regulation [34,43,44]. A subset of the miRNAs was associated with nervous system-specific processes, such as differentiation [45,46,47,48,49], lifespan [50], neurite guidance [51,52,53], or synaptogenesis [54,55,56]. We were specifically interested in miRNAs known to contribute to neuronal differentiation and/or axonal guidance, processes that are known to be regulated by RA [10,57]. One potential candidate was *miR-125b*, which contributes to the development of a permissive environment for axonal outgrowth in the injured CNS of axolotls [53]. However, this miRNA has not been shown to contribute to RA-induced regeneration in any species, indicating it was not a good candidate for further analysis.

Additional candidate miRNAs identified by our miRNA sequencing included *miR-124* and *miR-9*, both of which are enriched in the nervous systems of many species and contribute to neuronal differentiation [45,46,47,48,49] and axonal guidance [51,52]. Importantly, both of these miRNAs are upregulated by RA in human and mouse cell lines [47,58,59]. However, we found that *miR-9* was downregulated in RA-treated CNS (Figure 2C), indicating it may have distinct roles and responses to RA in the snail, compared to vertebrates. As described in vertebrates, however, *miR-124* was upregulated in RA-treated *Lymnaea* CNS (Figure 2C). As *miR-124* has been associated with neurite outgrowth, not only in mammals [60], but also in amphibians [52], we focused on examining a potential role for *miR-124* in regenerating neurons of the invertebrate, *Lymnaea*.

### 2.3. miR-124 Is Highly Enriched in the Adult Lymnaea CNS

**Figure 3:**
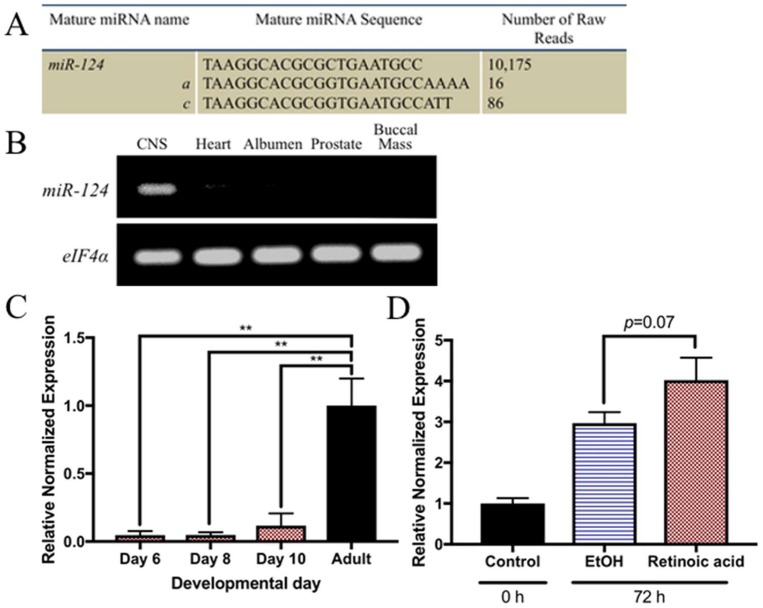
*miR-124* expression in the adult *Lymnaea* CNS. (**A**) Number of *miR-124* sequence reads in *Lymnaea* CNS, determined by miRNA sequencing. The family of *miR-124* contains *miR-124a* and *miR-124c* subtypes in low abundance; (**B**) Tissue-specific expression of *miR-124* in adult *Lymnaea*. PCR demonstrates that *miR-124* is enriched within the adult CNS, but appears diminished or completely undetectable in other tissues. *eIF4α* was used as the positive loading control; (**C**) *miR-124* expression during *Lymnaea* development. At 6, 8, and 10 days post-egg laying, *miR-124* expression remained relatively uniform, and was not statistically significant across developmental days. However, *miR-124* exhibited a significant increase in the adult CNS in comparison to earlier developmental days (** = *p* < 0.01); (**D**) *miR-124* expression in the regenerating *Lymnaea* CNS. The mean relative normalized expression is 26% greater in RA-treated CNS, in comparison to EtOH controls. However, this increase did not reach statistical significance (*p* = 0.07). For RT-qPCR, data was made relative to the expression of the acutely isolated CNS (control) and normalized to *β-tubulin*, *actin*, and *eIF4α*.

In vertebrates, *miR-124* has been characterized as a neuronally enriched miRNA [61] that contains multiple variants, including *miR-124a*, *miR-124b*, and *miR-124c* [62]. We obtained sequences for two conserved subtypes of *miR-124* in the *Lymnaea* CNS, *miR-124a* and *miR-124c*. However, the number of reads for each variant was notably low (Figure 3A). Rather than focus on each individual subtype, we instead characterized the entire *miR-124* family of microRNAs (henceforth referred to as *miR-124*).

PCR was used to determine whether *miR-124* shared a similar neuronally enriched expression in *Lymnaea* as it does in vertebrates. Indeed, we found *miR-124* was enriched in the CNS, but largely absent or undetectable from other adult tissues, including the heart, albumen, prostate, and buccal mass (Figure 3B). We next determined whether this miRNA was developmentally regulated, as it is in vertebrates. In vertebrates, *miR-124* is shown to temporally increase in abundance throughout development, until reaching maximal expression in the adult CNS [61,63,64]. To determine whether the same is true in *Lymnaea*, we performed qPCR on developing *Lymnaea* embryos and compared *miR-124* expression levels to that of the adult CNS. In developing *Lymnaea* embryos, the first neurons of the CNS are born approximately 4 days following egg laying [65]. In the days following, ganglia begin to form and continue to increase in size until ~10 days, when the embryos hatch [66]. As such, we examined *miR-124* expression at 6, 8, and 10 days after egg laying, at stages when the CNS has begun to develop [65,66]. *miR-124* was expressed at low levels over the course of *Lymnaea* development, and did not significantly increase in expression across these developmental time points (F_(3,8)_ = 17.63; *p* = 0.0007)(Figure 3C). However, *miR-124* is more abundant in the adult CNS, as shown in vertebrates [61,63,64] (Figure 3C), and was significantly higher than at 6 (*p* = 0.0013), 8 (*p* = 0.0014), and 10 days (*p* = 0.0022) of development.

After confirming *miR-124* was indeed a neuronally enriched miRNA in adult *Lymnaea*, we next used qPCR to confirm its differential expression between regenerating (RA-treated) and non-regenerating (EtOH-treated) CNS. Although the mean relative level of expression of *miR-124* in RA-treated brains was ~25% greater than in those treated with EtOH, this difference approached, but did not quite reach, statistical significance (*p* = 0.07; Figure 3D).

### 2.4. miR-124 Is Expressed in Both the Pedal and Right Parietal Ganglia

**Figure 4:**
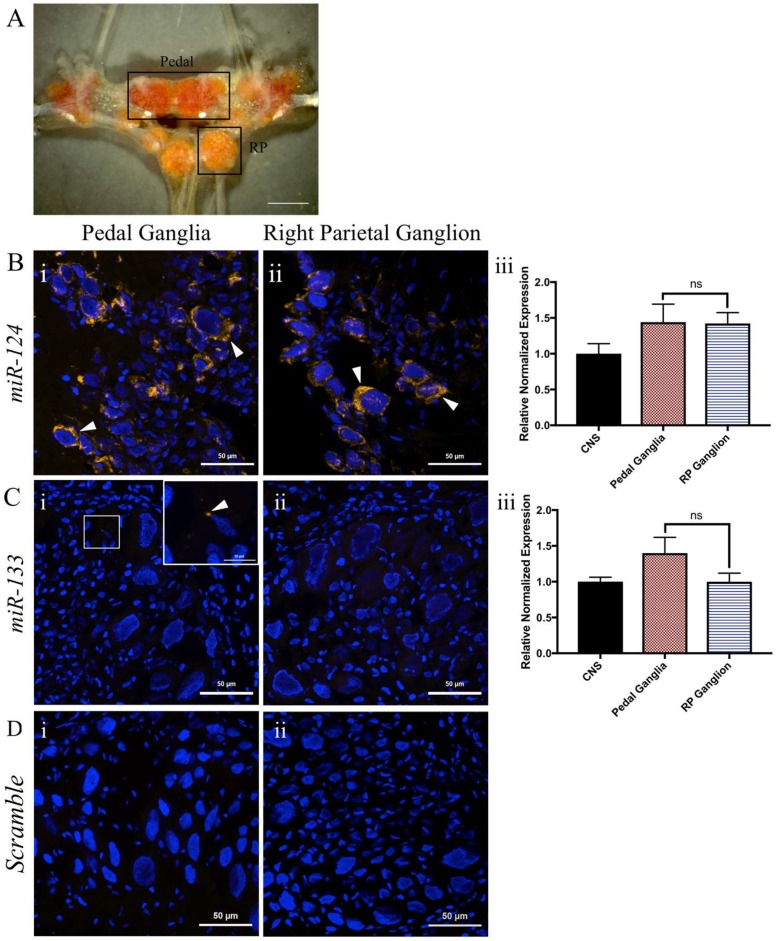
Expression of microRNAs *124* and *133* in different ganglia of the *Lymnaea* CNS. (**A**) Representative image of a *Lymnaea* CNS (Scale bar = 1 mm). Boxes represent different ganglia of the CNS, including the pedal ganglia and the right parietal (RP) ganglion; (**B**) *miR-124* demonstrates a perinuclear distribution in both the pedal (**Bi**) and right parietal (**Bii**) ganglia. Arrowheads indicate representative areas of expression. RT-qPCR analysis demonstrates uniform expression across both ganglia (**Biii**); (**C**) Little to no *miR-133* signal is detectable in either the pedal ganglia (**Ci**), or the right parietal ganglion (**Cii**). When examining *miR-133* utilizing RT-qPCR, we detected similar expression when comparing the pedal and right parietal ganglia, however, this expression was minimal. For RT-qPCR, data was made relative to the expression in the entire *Lymnaea* CNS, and normalized to *β-tubulin*, *actin*, and *eIF4α*; (**D**) Representative image of ganglia sections incubated with a scrambled probe (negative controls). No signal was detected in either pedal (**Di**) or right parietal (**Dii**) ganglia. Scale bars (**B**–**D**) = 50 μm.

miRNAs are generally cell type-specific and, importantly, have exhibited differential expression patterns in specific regions within the CNS of both mice [67] and zebrafish [68]. Our next aim was to determine whether specific patterns of expression of *miR-124* exist within different ganglia of the molluscan CNS. To this end, we utilized the pedal ganglia and right parietal ganglion (Figure 4A), which are known to contain different classes of motorneurons [69,70]. In situ hybridization indicated a perinuclear distribution of *miR-124* in cells of both the pedal ganglia (Figure 4(Bi)) and the right parietal ganglion (Figure 4(Bii)). Importantly, RT-qPCR analysis indicated that the overall expression levels of *miR-124* did not differ between these ganglia (*p* = 0.9535; Figure 4(Biii)). 

A different miRNA, *miR-133*, has previously been shown to regulate RA-induced regeneration of the newt spinal cord [14]. We thus also conducted in situ hybridization to examine its presence or absence in these same ganglia. No detectable signal was obtained for *miR-133* in either ganglion (Figure 4(Ci,Cii)). *miR-133* was, however, detectable by RT-qPCR (Figure 4(Ciii)), which showed no difference in expression across ganglia (*p* = 0.1862). These data also indicated very low expression levels of *miR-133*, confirmed also by our miRNA sequencing results. Hence, no further analysis was conducted for *miR-133*. 

### 2.5. miR-124 Is Expressed within the Cell Bodies and Neurites of Two Populations of Regenerating Motorneurons

We next examined *miR-124* expression patterns within cultured, regenerating motorneurons from both pedal and right parietal ganglia. This examination of cultured cells allowed a better resolution of cellular compartmentalization of the miRNA within regenerating neurons, and provided more detailed information on cell type-specific expression patterns. Importantly, different functional classes of motorneurons were used. Pedal A (PeA) motorneurons innervate the cilia of the foot musculature and are involved in locomotion [69]. These PeA ciliary motorneurons have previously been shown to exhibit robust outgrowth and chemotropic responses upon application of RA [10,18]. Another class of motorneuron, with as yet unknown responses to RA, were also included; these right parietal A (RPA) motorneurons control movement of the pneumostome and are, thus, required for aerial respiration [30]. 

**Figure 5:**
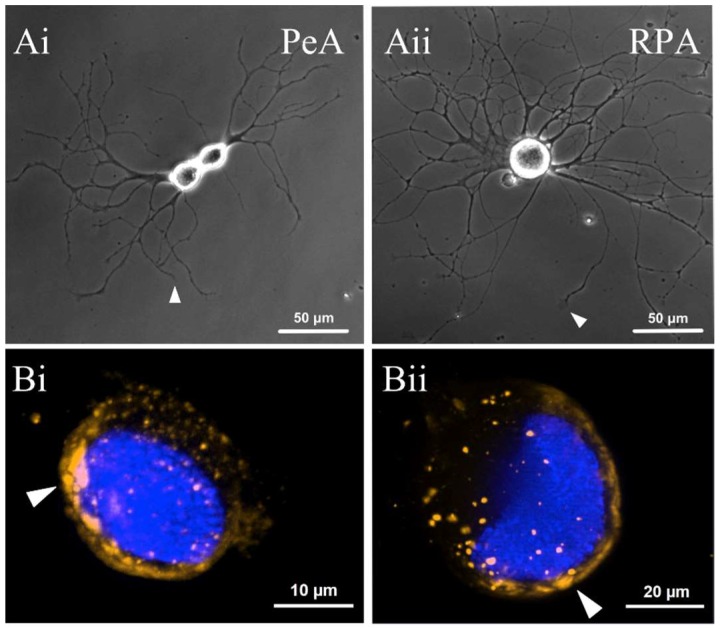

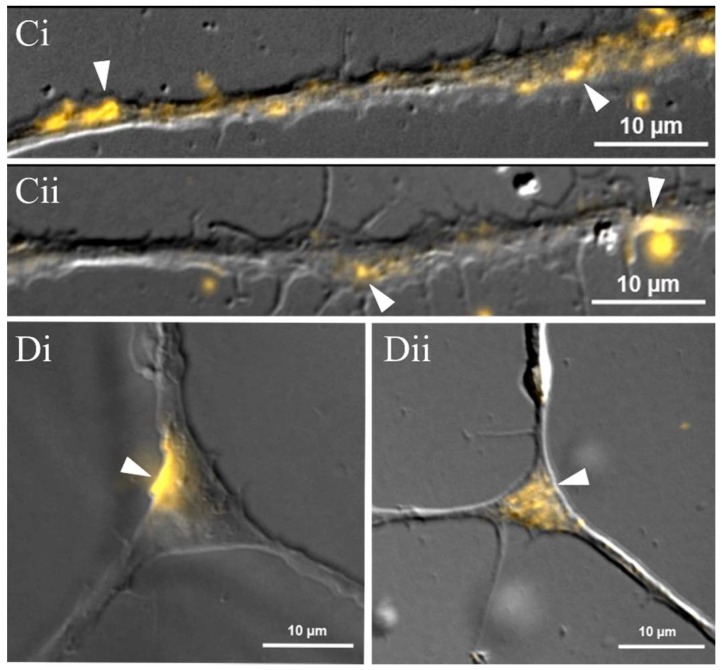
Expression of *miR-124* in cultured regenerating motorneurons. (**A**) Representative images of regenerating pedal A (PeA) (**Ai**) and right parietal A (RPA) (**Aii**) neurons in culture. In each cell, extending neurites contain multiple branch points and numerous growth cones. White arrows indicate growth cones at the tip of neurites; (**B**) *miR-124* exhibits a perinuclear distribution (indicated by white arrowheads) within the soma of regenerating PeA (**Bi**) and RPA (**Bii**) motorneurons; (**C**) Representative images of *miR-124* expression along a PeA (**Ci**) and RPA (**Cii**) neurite; (**D)** *miR-124* appears to be localized in many (but not all) branch points of PeA (**Di**) and RPA (**Dii**) neurites.

In the cultured regenerating motorneurons (Figure 5A), *miR-124* was consistently expressed within the cell bodies of both PeA (*n* = 46, Figure 5(Bi)) and RPA (*n* = 41, Figure 5(Bii)) cell types. Once again, *miR-124* clearly exhibited a perinuclear distribution within the soma, similar to the expression pattern we found in the ganglia. *miR-124* was also detected within regenerating neurites of both PeA (Figure 5(Ci)) and RPA motorneurons (Figure 5(Cii)). Interestingly, *miR-124* was expressed as individual punctae along the length of PeA and RPA neurites (Figure 5C). However, *miR-124* was not consistently expressed in all neurites of either cell type, with its expression in RPA neurites being less frequent in comparison to PeA neurites (Figure 5C). Interestingly, *miR-124* expression was often abundant in branch points of both PeA (Figure 5(Di)) and RPA (Figure 5(Dii)) neurites. 

### 2.6. miR-124 Is Differentially Expressed in the Growth Cones of Different Classes of Motorneurons

**Figure 6:**
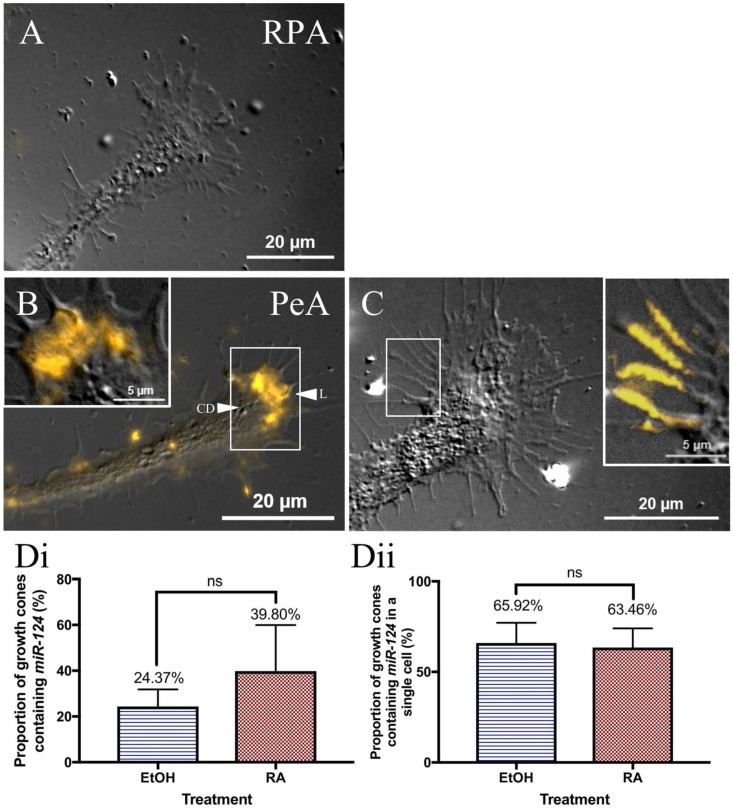
Differential expression of *miR-124* in motorneuron growth cones. (**A**) *miR-124* is not expressed in RPA growth cones (*n* = 0 of 43); (**B**) *miR-124* is expressed along the leading edge within the lamellipodia (L, arrowhead) of PeA growth cones, but is absent from the central domain (CD, arrowhead). White box indicates area represented by inset image, magnified to show *miR-124* in the lamellipodia; (**C**) *miR-124* is also expressed in the filopodia of PeA growth cones. White box indicates area represented by magnified inset image, demonstrating *miR-124* within filopodia; (**Di**) PeA cells incubated in 0.001% EtOH or 10^−7^ M RA do not exhibit a significant difference in the proportion of growth cones containing *miR-124* (*p* = 0.4995). ns = non-significant (**Dii**) When a PeA cell contains *miR-124* in one of its growth cones, over 60% of the cell’s remaining growth cones will also contain *miR-124* signal. This similar trend of expression is shown in cells incubated in both RA and EtOH. No statistical significance (ns) was detected between treatment groups (*p* = 0.8747).

Neuronal growth cones are structures responsible for initiating and guiding regenerative outgrowth. We, therefore, next examined the expression of *miR-124* within growth cones of the regenerating cultured RPA and PeA motorneurons. Interestingly, *miR-124* was not detected in the growth cones of any RPA neurons (*n* = 0 of 43 growth cones; Figure 6A). It was, however, expressed within growth cones of PeA neurons (*n* = 28 growth cones). *miR-124* was expressed along the leading edge of the PeA growth cones, and was most frequently restricted to the lamellipodia (L; Figure 6B). It was, however, noticeably absent from the central domain (CD) of the growth cones (Figure 6B). *miR-124* was also often associated or aligned with the filopodia in a long, fibrillar expression pattern (Figure 6C).

Interestingly, *miR-124* was not expressed in all PeA growth cones. Therefore, we next determined whether exposure of regenerating neurites to retinoic acid might affect the expression pattern and/or number of growth cones expressing *miR-124*. Cultured PeA motorneurons were incubated in either RA (10^−7^ M) or in EtOH (0.001%; vehicle–control) during the first 12–18 h of regenerative outgrowth. However, no significant differences in the proportion of growth cones containing *miR-124* were shown between treatment groups (*p* = 0.4995). *miR-124* was found in the growth cones of ~40% of RA-treated neurons and ~25% of EtOH-treated neurons (Figure 6(Di)).

Cultured PeA motorneurons typically contain multiple neurites with numerous growth cones (see Figure 5(Ai) as an example). With this in mind, we re-examined the proportion of growth cones on individual motorneurons expressing *miR-124*, this time, only analyzing those cells that expressed *miR-124* in at least one of its growth cones. Once again, the proportion of growth cones that expressed this miRNA did not differ between cells treated with either RA (~63%) or EtOH (~66%) (*p* = 0.8747; Figure 6(Dii)).

In summary, these data demonstrate cell type-specific expression of *miR-124* in *Lymnaea* growth cones, and that this pattern of expression is not dependent on prior exposure to RA. 

## 3. Discussion

In this study, we performed the first transcriptome analysis of miRNAs expressed during CNS regeneration in the invertebrate, *Lymnaea stagnalis*. *Lymnaea* is a useful model organism for the study of adult CNS regeneration due to its extensive regenerative capacity, and ease of isolation of large, identifiable neurons for cell culture. We identified 483 miRNAs in the adult *Lymnaea* CNS, and discovered a specific subset that may contribute to RA-induced regeneration. In particular, we focused on one neuronally enriched miRNA, *miR-124*. Using RT-qPCR, we confirmed the upregulation of *miR-124* during regeneration, and utilizing in situ hybridization, found that it was present within motorneurons. Interestingly, we found *miR-124* was enriched in the growth cones of PeA motorneurons, but was restricted to the cell bodies and/or neurites of RPA motorneurons. Together, these data are suggestive of a role for *miR-124* in RA-induced CNS regeneration. 

### 3.1. Lymnaea Stagnalis miRNA Transcriptome 

Using miRNA sequencing, we identified a large subset of 483 miRNA sequences in the snail CNS. Of these 483 identified sequences, 264 miRNAs were conserved in other molluscan species, while 219 represented a group of unique miRNAs that may be *Lymnaea*-specific. Interestingly, the number of novel miRNAs within the *Lymnaea* CNS was relatively high; representing 45% of all sequenced reads. In comparison, in the regenerating axolotl tail, fewer than 12% of the total sequences were identified as novel miRNAs [71]. Rather than reflecting an overabundance of novel *Lymnaea*-specific miRNA sequences, this finding may be due to the minimal sequence information available for the molluscan miRNA transcriptome. We expect that these potential novel miRNAs will be identified in other molluscan species as more sequence data becomes available. 

Interestingly, many of these novel sequences were differentially regulated during CNS regeneration. Specifically, 86 novel miRNA sequences exhibited at least a 2-fold increase or decrease in RA-treated CNS, corresponding to 39% of all the novel sequences. We found that many of the known or characterized mature miRNAs that were differentially regulated contributed to similar biological processes, including neuronal differentiation, proliferation, neurite guidance, or synaptogenesis. As such, we predict the differentially regulated novel sequences may contribute to similar events during CNS regeneration. However, a majority of the novel miRNAs exhibited an extremely low number of reads, generally much lower than *miR-133* (which was undetectable by in situ hybridization). With such low abundance, characterizing the specific functions of these novel sequences will prove to be difficult, as they may be undetectable by most standard molecular techniques. 

### 3.2. miR-124 Expression Patterns in Lymnaea CNS

Using both miRNA sequencing and RT-qPCR analyses, we found *miR-124* was abundant within the adult *Lymnaea* CNS, and was upregulated in regenerating CNS, implicating its potential role in molluscan CNS regeneration. *miR-124* is a well-characterized miRNA that is predominantly expressed in neuronal cells, and regulates a variety of processes, including neuronal differentiation [47,48], neurite outgrowth [52], neuronal cell fate [47,61], and the transition from neural progenitors to mature neurons [47]. Importantly, *miR-124* has also been shown to regulate CNS regeneration in flatworms of the class Turbellaria [72]. When *miR-124* was inhibited during planarian brain regeneration, this resulted in a significant reduction of dopaminergic and GABAergic neurons, reducing the overall brain size [72]. Together with our data, these studies support a role for *miR-124* in the regeneration of invertebrate nervous systems.

In mice, *miR-124* expression is 100 times higher in the CNS than in other tissues [73], and gradually increases in abundance in parallel with neuronal maturation [61]. We found similar trends in *Lymnaea*, as *miR-124* was enriched in the CNS, compared to other tissues and organs. Moreover, we also discovered *miR-124* exhibited very low levels of expression during *Lymnaea* development, but was highly enriched in the mature adult snail CNS. Collectively, these data indicate *miR-124* shares similar expression patterns in an invertebrate species as it does in some vertebrates, and is highly enriched in the mature CNS. 

### 3.3. Expression of miR-124 in Motorneurons

To further characterize *miR-124* expression, we determined the subcellular distribution of *miR-124* within individual regenerating motorneurons, and specifically, within the growth cone. The presence of miRNAs within neuronal growth cones has previously been indicative of their role in controlling neurite outgrowth and growth cone guidance [52,74,75,76]. As such, miRNAs within the growth cone can rapidly downregulate mRNAs that might impede neurite sprouting or impede specific turning responses. In *Xenopus laevis*, *miR-124* is localized to the growth cones of retinal ganglion cells, and regulates neurite outgrowth in response to the guidance cue, Sema3A [52]. Similarly, in our present study, we discovered that *miR-124* was expressed in the growth cones of PeA ciliary motorneurons. Interestingly, we determined that the number of PeA growth cones containing *miR-124* was not significantly altered when cells were cultured in the presence of RA in comparison to EtOH alone. This may indicate that *miR-124* does not play an integral role in RA-induced neurite sprouting. However, *miR-124* may instead contribute to fast-acting growth cone turning responses, similar to other vertebrate growth cone-specific miRNAs [52,74,75,76]. Within vertebrate growth cones, miRNAs have been shown to regulate local protein synthesis to rapidly alter cytoskeleton dynamics in response to specific guidance cues. In *Lymnaea*, when local protein synthesis is inhibited in PeA growth cones, their attractive chemotropic response to RA is abolished [18]. This may implicate *miR-124* as a positive regulator of local mRNA translation during fast-acting growth cone turning responses, as opposed to a substantial role in neurite sprouting.

Interestingly, we discovered that *miR-124* did not share a similar expression pattern in the growth cones of RPA motorneurons. *miR-124* was expressed in ~40% of all PeA growth cones, but completely absent in all RPA growth cones examined. This suggests that *miR-124* may not be involved in regulating growth cone guidance in all motorneuron cell types. Indeed, it is likely that different miRNAs may mediate the turning behaviors of different classes of motorneurons, possibly in response to a variety of guidance cues encountered during innervation of different targets during development.

In this study, we detected *miR-124* in the cell bodies of both populations of *Lymnaea* motorneurons studied, though, interestingly, it was not previously detected in the motorneurons of another molluscan species. In *Aplysia californica*, *miR-124* was found to be an essential regulatory molecule at the cultured sensory-motor synapses, where it regulated the transcription factor, CREB [56]. *miR-124* was, however, primarily expressed in the sensory neurons and was undetected in the motorneurons [56]. However, the sensory and motorneurons were cultured together, and formed synaptic connections [56], which may have affected expression levels in either cell, compared to neurons cultured in isolation. It is possible that *miR-124* may exhibit a higher abundance in motorneurons prior to detecting a synaptic partner. Alternatively, the expression of *miR-124* may be species-specific and/or cell type-specific. Due to the limited number of sensory neurons that have been identified in *Lymnaea*, we did not compare the expression patterns of this miRNA between sensory and motorneurons in this study.

### 3.4. Role of miR-124 during RA-Induced CNS Regeneration

Using miRNA sequencing, we demonstrated that *miR-124* was upregulated in RA-treated CNS, a trend that has also been described in vertebrate cell cultures [58,59]. In *Lymnaea*, the expression of both nuclear receptors that bind retinoic acid, RXR [19] and RAR [20], increase during CNS development. Similarly, a specific RAR subtype, RARβ, has been shown to increase in a stage-specific manner during CNS regeneration in the adult newt [13]. As both RXR and RAR are likely critical during RA-induced regeneration [13,77], and expressed in *Lymnaea* PeA growth cones [19,20], it is possible that *miR-124* could be targeting either mRNA sequence during specific stages of regeneration. Indeed, the 3′UTR of the *Lymnaea RAR* and *RXR* mRNAs contain potential binding sites for *miR-124*, though we have not yet explored whether these mRNAs are found locally in the neurites and/or growth cones. Interestingly, during newt spinal cord regeneration, RARβ protein increases [13], while RXR is instead downregulated [77]. If similar trends are exhibited during invertebrate CNS regeneration, it is feasible that *Lymnaea RXR* may act as a potential target for *miR-124* during *Lymnaea* CNS regeneration. 

Alternatively, *miR-124* may target mRNAs responsible for degradation of RA. Specifically, Cytochrome P450 protein 26 (Cyp26) is responsible for degrading all-*trans* RA, and is expressed in *Lymnaea* (Genbank Accession No. KF669878). Indeed, the 3′UTR of the *Lymnaea Cyp26* mRNA contains multiple binding sites for *miR-124*, suggesting this mRNA may also act as a potential binding site for *miR-124* during RA-induced regeneration. However, its presence within PeA or RPA neurites and growth cones has not yet been determined. Alternatively, *miR-124* may target mRNAs that may impede neuronal outgrowth. However, as the *Lymnaea* genome has not yet been sequenced, it is difficult to obtain an exhaustive list of potential *Lymnaea* mRNA targets for *miR-124* at this time.

In summary, this study provides the first miRNA sequencing analysis of miRNAs in the regenerating CNS of the mollusc, *Lymnaea stagnalis* and, more importantly, characterizes classes of both novel and conserved miRNAs that are regulated during RA-induced regeneration of the adult CNS in this invertebrate. We also demonstrate that a specific, conserved miRNA, *miR-124*, is abundant in the adult snail CNS, as it is in vertebrates. Furthermore, we provide new evidence for cell type-specific expression of *miR-124* in the growth cones of different classes of motorneurons. In future studies, it will be important to characterize the expression patterns and potential targets of our other miRNAs whose expression is mediated by RA signaling, and to identify mRNA targets and functions of these miRNAs during CNS regeneration.

## 4. Methods

### 4.1. Isolation of CNS 

*Lymnaea stagnalis* were bred in the laboratory and kept in aerated pond water at room temperature on a 12 h/12 h light–dark cycle. For all experimental procedures, adult snails were anesthetized in 25% Listerine^®^ (containing menthol; 0.042% *w*/*v*, Johnson & Johnson Inc., Markham, ON, Canada) in pond water prior to removal of the central ring ganglia (CNS). 

### 4.2. Regenerating CNS Preparation

Isolated *Lymnaea* CNS were incubated in 3 mL of defined medium (DM; comprised of 50% Leibovitz’s L-15 (Gibco, Dublin, Ireland) and additional salts) containing 10^−5^ M RA (Sigma, Oakville, ON, Canada) (to induce neural regeneration/neurite sprouting) in a plastic Falcon dish (VWR, Radnor, PA, USA) for 72 h. Different CNS were also incubated in 0.1% EtOH (Greenfield Global, Brampton, ON, Canada) as a vehicle control. Following the 72 h incubation period, any neurite sprouting from individual nerves emanating from the CNS were imaged using Q-Capture imaging software (v2.90.1, Q Imaging, Surrey, BC, Canada). 

### 4.3. RNA Sequencing of Lymnaea miRNAs

RA-treated (regenerating) and EtOH-treated (non-regenerating) samples were collected by pooling five-CNS from adult *Lymnaea* (shell length of 20–25 mm) per sample (1 biological replicate was utilized). Total RNA was extracted using TRI Reagent (Sigma) and Direct-zol RNA MiniPrep kit (Zymo Research, Irvine, CA, USA), and outsourced to LC Sciences (Houston, TX, USA) for deep sequencing. A small RNA library was generated from both regenerating and non-regenerating CNS samples using the Illumina Truseq™ Small RNA Preparation kit (Illumina Inc., San Diego, CA, USA), according to the manufacturer’s instructions. This generated library was utilized for cluster generation on Illumina’s Cluster Station and sequencing on Illumina GAIIx (Illumina). Raw sequencing reads were obtained using Illumina’s Sequencing Control Studio software version 2.8 (SCS v2.8) following real-time sequencing image analysis and base-calling by Illumina Real-Time Analysis version 1.8.70 (RTA v1.8.70, San Diego, CA, USA). 

### 4.4. Isolation of Lymnaea Embryos

Egg masses were incubated in pond water at room temperature for 6, 8, and 10 days post-egg laying, corresponding to various stages of *Lymnaea* development (as described by Nagy and Elekes [66]). At each developmental stage (day 6, 8, and 10), egg capsules were removed from their gelatinous surroundings. Following isolation, all embryos encased in one egg mass were pooled and frozen on liquid nitrogen for molecular analysis. 

### 4.5. Cell Culture

Adult snails (16 to 20 mm in length) were used for all cell culture procedures. Following isolation of the *Lymnaea* CNS, individual ganglia were desheathed to expose cells of interest, including pedal A (PeA) and right parietal A (RPA) motorneurons. Individually identified neurons were removed from the ganglia using suction applied via a fire polished pipette, and then plated on poly-l-lysine (Sigma)-coated Falcon dishes (VWR). Culture dishes contained 3 mL of conditioned medium (CM), which contain unidentified trophic factors that can produce neurite outgrowth [18,78]. In addition, 10^−7^ M RA was added to the culture dishes overnight to promote neurite sprouting [10,17]. All culture dishes were maintained at 21 °C overnight. To determine the effects of RA on the proportion of growth cones expressing *miR-124*, cells were cultured in either 10^−7^ M RA or 0.001% EtOH (as the vehicle control). 

### 4.6. RNA Isolation and cDNA Synthesis

All *Lymnaea* organs and tissues were isolated from adult *Lymnaea stagnalis* with a shell length of 20–25 mm, then immediately flash frozen in liquid nitrogen. For molecular analyses, one pooled sample for each organ contained: 2 CNS, 10 hearts, 10 albumen organs, 4 prostates, or 4 buccal masses. For analysis of individual CNS ganglia, the following were pooled for each sample: 20 pedal ganglia, or 31 right parietal ganglia. For regenerating CNS samples, each biological replicate contained five pooled CNS. For all experiments, 3 biological replicates were utilized. 

Total RNA was isolated from these samples using TRI Reagent (Sigma) and Direct-zol RNA MiniPrep kit (Zymo Research). RNA quality was confirmed using spectrophotometry and gel electrophoresis. A total of 750 ng of RNA was utilized from each sample for cDNA synthesis using gene specific stem-loop primers with the SuperScript III Reverse Transcriptase kit (Invitrogen, Burlington, ON, Canada). Stem-loop primers were designed for *miR-124* (RT: GTCGTATCCAGTGCAGGGTCCGAGGTATTCGCACTGGATACGACGGCATT) and *miR-133* (RT: GTCGTATCCAGTGCAGGGTCCGAGGTATTCGCACTGGATACGACAGCTGG) based on sequences provided by miRNA sequencing.

### 4.7. PCR

PCR reactions contained a total volume of 20 μL, consisting of 10× Taq buffer (Bio-Rad, Mississauga, ON, Canada), 50 mM MgCl_2_ (Bio-Rad), 10 mM dNTP mix (10 mM each of dATP, dGTP, dCTP, and dTTP) (Bio-Rad), iTAQ (Bio-Rad), both forward and reverse primers (10 μM), template cDNA (750 ng), and nuclease-free water. Forward and reverse primers were as follows for *miR-124*: (F: GCCGCTAAGGCACGCG GTG; R: GTGCAGGGTCCGAGGT), *miR-133*: (F: GCCGCTTTGGTCCCCTTCA; R: GTGCAGGGTCCGAGGT), and *eIF4α* as a positive control: (F: CCGAGCGTGTAAGC AGCC; R: AGTTGTGGTTGACTTGCCAGAG). Thermal cycling parameters included an initial DNA denaturation step at 95 °C for 3 min, then temperature cycling of 95 °C for 30 s (denaturation), 55 °C for 30 s (primer annealing), and 72 °C for 1 min (polymerase extension), and repeated for 40 cycles. End products combined with DNA loading dye were run on a 2.5% agarose gel and visualized using ethidium bromide staining.

### 4.8. RT-qPCR

Following cDNA synthesis, RT-qPCR was run using iQ SYBR Green Supermix (Bio-Rad) utilizing a CFX Connect Real-Time System (Bio-Rad). All samples were incubated for an initial DNA denaturation step for 3 min at 95 °C, then underwent 40 thermocycles of 95 °C for 30 s (DNA denaturation), 55 °C for 1 min (primer annealing) and 72 °C for 1 min (polymerase extension). Samples are reported relative to acutely isolated CNS, and normalized to *β-tubulin* (F: CATCCCCTAGCCATCTCTTCA; R: AGAGAGGCCTGGAGAGCTAA), *actin* (F: GCGATCTCACCGACTACCTG; R: ACGGACAATCTCACGCTCAG), and *eIF4α* (F: CCGAGCGTGTAAGCAGCC; R: AGTTGTGGTTGACTTGCCAGAG). Semi-quantitative analysis was performed utilizing the ΔΔ*C*_T_ method, in accordance with the MIQE guidelines. 

### 4.9. LNA-FISH and Tyramide Signal Amplification

Cultured cells or CNS were fixed in 4% paraformaldehyde (Sigma) for 20 min at room temperature, then stored at 4 °C in phosphate-buffered saline (PBS; Sigma) until required. Prior to staining procedures, both cells and CNS were treated with 3% H_2_O_2_ (Sigma) for 1 h at room temperature to remove endogenous peroxidase activity. 

CNS were next washed, on rotation, with increasing sucrose concentrations: 10% for 30 min, 20% for 30 min, and 30% overnight at 4 °C. Samples were then embedded in Optimal Cutting Temperature (O.C.T.) compound (Tissue-Tek, Sakura, Osaka, Japan), and 12 μm sections were obtained using a cryostat (Leica Microsystems, Richmond Hill, ON, Canada). Tissue sections were mounted on Superfrost Plus slides (Fisher Scientific, New Hampshire, NH, USA). 

For staining procedures, CNS tissue sections and cultured cells underwent the same procedures modified from Lu and Tsourkas [79]. Samples were first washed in PBS, then dehydrated overnight at 4 °C in 70% ethanol. The next day, tissue sections and cells were incubated in hybridization buffer (25% formamide (Sigma), 0.05M EDTA (Sigma), 4× saline-sodium citrate (SSC) buffer (Sigma), 10% dextran sulfate (Sigma), 1× Denhart’s solution (Sigma), 0.5 mg/mL *E. coli* tRNA (Sigma), 20 mM ribonucleoside vanadyl complexes (Sigma), 9.2 mM citric acid (Sigma)) at 58 °C (for *miR-124* and scrambled probe) or 52 °C (for *miR-133*) for 2 h, then hybridized in 10 nM of probe (Exiqon, Woburn, MA, USA) for 1 h at the same temperature. Samples next underwent stringency washes with decreasing concentrations of SSC, including 4× SSC briefly, 2× SSC for 30 min, 1× SSC for 30 min, and 0.1% SSC for 20 min. Following these washes, cells and tissues were incubated in blocking buffer (3% bovine serum, 4× SSC, 0.1% Tween-20) for 30 min, then horseradish peroxidase (HRP) (Sigma) for 30 min at room temperature. Samples were next washed in TNT buffer (0.1 M Tris HCl, 0.15 M NaCl, 0.05% Tween-20), then incubated in tyramide for 1 h at room temperature. Following a final series of washes in TNT buffer, all samples were mounted and imaged using a Carl Zeiss Axio Observer.Z1 inverted light/epifluorescence microscope, with ApoTome.2 optical sectioning (Carl Zeiss Canada Ltd., North York, ON, Canada). 

### 4.10. Statistics

All data were analyzed utilizing GraphPad Prism, Version 7.0 for Mac OS X (La Jolla, CA, USA), and values were expressed as the mean ± SEM. For statistical analysis investigating *miR-124* expression during *Lymnaea* development, a one-way analysis of variance (ANOVA) was performed, followed by Tukey’s post hoc test. For all other statistical analyses, a Students unpaired *t*-test was performed. For all analyses, a *p value* less than 0.05 was considered to be statistically significant. 

## Figures and Tables

**Table 1:** Putative biological roles and mRNA targets of miRNAs that exhibited differential expression following treatment with RA.

**Biological Role**	**Mature miRNA**	**Target**	**Reference**
Tumorigenesis	*let-7*	*Cdk4*	[23]
		*HMGA2*	[24]
	*miR-96*	*FOXO1*	[25]
	*miR-124*	*Cdk4*	[26]
	*miR-125*	*ErbB2*	[27]
		*ENPEP*, *CK2-α*, *CCNJ*, *MEGF9*	[28]
	*miR-182*	*BRCA1*	[29]
	*miR-193*	*ERBB4*	[30]
Differentiation	*miR-7*	*RAS*	[31]
	*miR-29b*	*FBXO2*	[32]
	*miR-184*	*Saxophone*	[33]
Proliferation	*Bantam*	*Hid*	[34]
	*miR-96*	*PTPN9*	[35]
	*miR-125*	*A20*	[36]
	*miR-182*	*FOXF2*	[37]
	*miR-184*	*Numbl*	[38]
Apoptosis	*miR-9*	*MTHFD2*	[39]
	*miR-29*	*FoxM1*	[40]
	*miR-96*	*FOXO1*	[25]
	*miR-182*	*FOXO1*	[41]
	*miR-193*	*MCL1*	[42]
Cell cycle regulation	*let-7*	*Cdc34*	[43]
	*Bantam*	*hid*	[34]
	*miR-182*	*c-Met*	[44]
**Nervous System-Specific Role**	**Mature miRNA**	**Target**	**Reference**
Neuronal differentiation	*miR-9*	*PTBP1*	[45]
		*Rap2a*	[46]
	***miR-124***	*PTBP1*	[47]
		*Rap2a*	[46]
		*Sox9*	[48]
		*JAG1*	[49]
Neuronal lifespan	*let-7*	*Chinmo*	[50]
	*miR-125*	*Chinmo*	[50]
Neurite guidance	*miR-9*	*Gsh2*, *Foxg1*	[51]
	***miR-124***	*coREST*	[52]
	*miR-125*	*Sema4d*	[53]
Synaptogenesis	*miR-8*	*Fasciclin III*, *Neuroglian*	[54]
	***miR-124***	*GluA2*	[55]
		*CREB*	[56]

## Supplementary Materials

Supplementary materials can be found at http://www.mdpi.com/1422-0067/19/9/2741/s1.
